# Adenovirus-mediated delivery of CALR and MAGE-A3 inhibits invasion and angiogenesis of glioblastoma cell line U87

**DOI:** 10.1186/1756-9966-31-8

**Published:** 2012-02-01

**Authors:** Xin-Li Liu, Dan Zhao, Da-Peng Sun, Yang Wang, Yan Li, Feng-Qi Qiu, Ping Ma

**Affiliations:** 1 Cancer Research Institute, First Affiliated Hospital, China Medical University, Shenyang 110001, China; 2 Cancer Research Institute, First Affiliated Hospital, China Medical University, Shenyang 110001, Liaoning Province, China

## Abstract

**Background:**

The management of patients with glioblastoma multiforme is difficult. Poor results have led to a search for novel therapeutic approaches. Gene therapy that could be both anti-invasive and antiangiogenic would be ideal. In this study, we constructed the recombinant adenoviral vector Ad-CALR/MAGE-A3 and evaluated its antitumor effects on glioblastoma in vitro and in vivo.

**Methods:**

In this study, CALR and MAGE-A3 genes were delivered to the glioblastoma cell line U87, using adenovirus (Ad-CALR/MAGE-A3). U87 glioblastoma cells were transfected with Ad-green fluorescent protein to identify the multiplicity of infection. The expressions of CALR and MAGE-A3 were detected by PCR and Western blot. Cell proliferation was measured by MTT assay. Cell apoptosis was assessed by Annexin-V FITC/PI double staining flow cytometry. The invasive potential of U87 cells was determined by Matrigel invasion assay. Tube formation assay was used to detect the effects on angiogenesis of human umbilical vein endothelial cells. Protein expressions of PI3K/AKT, Erk1/2 and MMP-2/-9 in transfected cells were detected by Western blot. In vivo, the effects of Ad-CALR/MAGE-A3 on tumor growth and angiogenesis of U87 glioblastoma xenografts in nude mice were investigated.

**Results:**

The expressions of CALR and MAGE-A3 in U87 cells resulted in the suppression of cell proliferation and invasion properties, and induced cell apoptosis. The Erk MAPK, PI3K/AKT pathways and expressions of MMP-2/-9 were inhibited in Ad-CALR/MAGE-A3-transfected cells. Outcomes of the tube formation assay confirmed the antiangiogenic effect of CALR. Moreover, in the in vivo model of glioblastoma, intratumoral injection of Ad-CALR/MAGE-A3 suppressed tumor growth and angiogenesis.

**Conclusion:**

Although Ad-CALR/MAGE-A3 and Ad-CALR demonstrated antiangiogenic effects on U87 cells, the repression of invasion was significant only in Ad-CALR/MAGE-A3-treated cells. To our knowledge, this is the first description of a role for combined CALR and MAGE-A3 in the anti-invasion and antiangiogenesis of U87.

## Background

The most frequent form of brain tumor in adults is glioma [1]. Types of gliomas include astrocytomas, oligodendrogliomas, oligoastrocytomas, and ependymomas [2]. Astrocytoma is the most common, and on the World Health Organization's international classification of human tumors scale, astrocytomas may carry a histological grade anywhere from I (low proliferative potential and the possibility of cure) to IV (cytologically malignant, mitotically active, and typically fatal). By contrast, oligodendrogliomas and oligoastrocytomas are usually classified either grade II or III [3].

The grade IV astrocytic tumor, or glioblastoma, is highly invasive and clinically challenging. Despite application of multimodal therapies, median survival is only 12-15 months [4]. There is a tremendous need to develop novel approaches to treat glioblastoma, and virus-mediated gene therapy is a viable possibility. A novel gene therapy that could achieve an antiangiogenic and anti-invasive effect would reduce the tumor's vascular permeability and prolong progression-free survival, and is therefore critically important.

Melanoma antigen gene-A3 (MAGE-A3) is a cancer-testis antigen. Its expression in normal tissues is limited to the testes but it is found at high levels in various tumors [5-7]. Indeed, immunotherapeutic trials targeting MAGE peptides have achieved encouraging results in patients with metastatic melanoma [8-10]. However, there is currently limited evidence implicating MAGE-A3 activity in cancer progression. Other MAGE-A gene members, such as MAGE-A4, have been reported to promote apoptosis in non-small cell lung cancer [11], and MAGE-D1 may be a novel endogenous inhibitor of angiogenesis in vitro and in vivo [12]. The putative functions of MAGE family members highlight the importance of their detailed characterization with regard to cancer progression.

Calreticulin (CALR) is an abundant 46-kDa Ca^2+^- binding protein which was first located in the endoplasmic reticulum [13,14], but is also found at the cell surface and nucleolus [15,16]; it performs a variety of functions within the cell [17-19]. Although the role of CALR in normal cellular functions and embryogenesis is well-established, the parts it plays in human carcinogenesis are poorly understood [20]. It has been reported to act as an endothelial cell inhibitor of tumor growth and its chaperone effect in cancer vaccines was also shown [21,22]. Recently, the repressive effect of CALR on tumor invasion, including that of the prostate [23], has become a popular field of research.

Adenovirus-based transfer of a gene into cells causes a transient spike in the levels of the protein the gene encodes. The technique reduces the possibility of experimental error to some extent. To the best of our knowledge, no prior study has attempted the simultaneous adenovirus-mediated gene transfer of the genes CALR and MAGE-A3 (Ad-CALR/MAGE-A3) to evaluate their combined antitumor effect or antitumor mechanism in glioblastoma. In this study, we successfully used Ad-CALR/MAGE-A3 to express CALR and MAGE-A3 proteins in the glioblastoma cell line U87. In both in vitro and in vivo experiments we demonstrate that tumor growth and invasive abilities are reduced, while apoptosis is induced, in Ad-CALR/MAGE-A3-transfected U87 cells. In addition, molecular mechanisms underlying the antitumor effects of Ad-CALR/MAGE-A3 are partially revealed, which could serve as a rationale for gene therapy in the treatment of glioblastoma.

## Methods

### Cell lines and cell culture

Cells of the human embryo kidney cell line 293-LP and human glioblastoma cell line U87 were grown in Dulbecco's modified Eagle's medium (DMEM), supplemented with 10% fetal bovine serum. Human umbilical vein endothelial cells (HUVECs) were grown in Kaighn's modification of Ham's F-12 medium (F-12 K), with 0.1 mg/mL heparin, 0.03-0.05 mg/mL endothelial cell growth supplement, and 10% fetal bovine serum (FBS), in a humidified atmosphere containing 5% CO_2 _at 37°C. All cells were purchased from the Institute of Biochemistry and Cell Biology, Shanghai Institute for Biological Sciences, Chinese Academy of Sciences. All media and sera were purchased from Gibco.

### Adenoviral vector construction and transfection

To create Ad-CALR, a fragment of CALR was excised using EcoRI/KpnI and cloned into a pShuttle- green fluorescent protein (GFP)- cytomegalovirus (CMV) plasmid to produce the shuttle vector. CALR was subsequently excised from the shuttle vector using I-CeuI and I-SceI and ligated into the pAd vector for the recombinant generation of Ad-CALR. To create Ad-CALR/MAGE-A3, a fragment of CALR was excised using NheI/PmeI and cloned into a pShuttle-GFP-CMV plasmid; a fragment of MAGE-A3 was excised by BglII/XhoI and cloned into the pShuttle-(ΔGFP)-CALR plasmid. CALR/MAGE-A3 was subsequently excised from the shuttle vector using I-CeuI and I-SceI and ligated into the pAd vector for the recombinant generation of Ad-CALR/MAGE-A3. Ad-CALR and Ad-CALR/MAGE-A3 were further amplified in HEK293LP cells. Viral particles were purified using cesium chloride density gradient centrifugation. 293-LP cells in serum-free DMEM were transfected with Ad-GFP to identify the optimal conditions. U87 cells (2 × 10^6^) were transfected with Ad-vector, Ad-CALR, and Ad-CALR/MAGE-A3 at 100 multiplicity of infection (MOI), (calculated as the number of plaque-forming units [PFU] per cell), in a humidified atmosphere containing 5% CO_2 _at 37°C. Transfection with a null plasmid served as a control. The cells were harvested 48 h after transfection for analyses.

### Reverse transcription-PCR and real-time quantitative RT-PCR (qRT-PCR)

All PCR kits were purchased from Takara, Japan. Total RNA was isolated from cultured cells using an RNAiso Plus kit (1 mL per 5 × 10^6 ^cells). The concentration and purity of RNA were detected by an ultraviolet spectrometer. cDNA was generated according to the RNA reverse transcription (RT) kit instructions. MAGE-A3 fragment was amplified with forward primer 5'-CTGCTCACCCAACATTTCGT-3', reverse primer 5'-CACTCTTCCCCCTCTCTCAA-3'. MAGE-A3/PolyA fragment was amplified with the forward primer and reverse primer of PolyA 5'-GTGGTTTGTCCAAACTCATCAA-3'. PCR conditions were: 95°C for 15 min; 30 cycles of 94°C for 30 s, 55°C for 30 s, and 72°C for 2 min; and 4°C hold. Ten microliters of PCR product was analyzed on 2% agarose gels. SYBR^® ^Premix Ex Taq™ (Perfect Real Time) was used for real-time PCR (qPCR) of CALR. The Light Cycler PCR system (Roche Diagnostics, Mannheim, Germany) was used for qPCR amplification and cycle threshold (Ct) detection. The thermal cycling conditions comprised an initial denaturation step at 95°C for 30 s, 40 cycles at 95°C for 5 s, and 61°C for 30 s. Primers were 5'-GCACTTGGATCCACCCAGAA-3' and 5'-GAAGTTGTCAAAGATGGTGCCAGA-3'. The melting curves were analyzed after amplification. Each PCR reaction was done in triplicate.

Relative changes in expression were calculated using the 2^-ΔΔ*Ct *^method (Reference), where Δ*Ct *is the difference in threshold cycles for the target gene and reference (ACTB), and ΔΔ*Ct *is the difference between the Δ*Ct*s of the treated sample and control or calibrator. Thus, the expression levels were reported as fold changes relative to the calibrator. The value was used to plot the expression of apoptotic genes with the formula 2^-ΔΔCt^.

### Western blot analysis

Four sub-group U87 cells were lysed in radioimmunoprecipitation (RIPA) buffer and total protein concentration was determined with a bicinchoninic acid (BCA) assay (Beyotime, China). Twenty micrograms of total protein were separated by 10% SDS-PAGE and then transferred to polyvinylidene fluoride membranes. The membranes were washed, blocked, and incubated sequentially with specific primary antibodies, namely: rabbit monoclonal anti-CALR (1:1000), rabbit polyclonal anti-MAGE-A3 (1:100), both from Abcam (MA, USA); anti-PI3K (1:200), anti-Akt (1:200)/phosphorylated (p)-Akt (1:200), anti-Erk1/2 (1:200)/p-Erk1/2 (1:200) from Santa Cruz (CA, USA); mouse monoclonal anti-matrix metalloproteinases (MMP)2 (1:1000), rabbit monoclonal anti-MMP9 (1:10000) and rabbit polyclonal anti-β-actin (1:1000) from Abcam. Incubation in primary antibodies was followed by horseradish peroxidase -conjugated anti-rabbit secondary antibody (Zhongshan, 1:2000).

The reactions were detected by enhanced chemiluminescence assay. Each experiment was performed in triplicate.

### Cell proliferation assay

Cell proliferation was detected by methyl-thiazolyl-tetrazolium (MTT) assay. U87 cells were seeded in 96-well plates at a density of 1 × 10^4 ^cells/well. After 24 h, the cells were transfected with null, Ad-vector, Ad-CALR or Ad-CALR/MAGE-A3 and cultured for 1-7 d. Cell proliferation was determined by adding MTT (5 mg/mL) and incubating the cells at 37°C for 4 h further. The precipitate was solubilized by the addition of 150 μL/well dimethyl sulfoxide (Sigma) and shaken for 10 min. Absorbance at a wavelength of 490 nm was measured in each well with a microplate reader (Bio-Tek ELX800, USA).

### In vitro invasion assay

Invasion assays were performed using a 24-well plate invasion chamber (Corning, USA) fitted with cell culture inserts, and closed with 8 μm pore-size poly(ethylene terephthalate) (PET) membranes coated with a thin layer of Matrigel basement membrane matrix (BD Matrigel™). The lower chamber was filled with 600 μL DMEM supplemented with 10% FBS added as a chemoattractant. In the upper chamber, 100 μL of cells previously grown in DMEM for 12 h were seeded at 2 × 10^5 ^cells/mL in serum-free medium. The total number of cells that had migrated to the underside of the membranes after 48 h was counted under a light microscope in five predetermined fields (×100) after fixation and staining with crystal violet. All assays were independently repeated ≥ 3 ×.

### Flow cytometric analysis of apoptosis

Apoptosis was examined by using an fluorescein isothiocyanate (FITC) Annexin-V Apoptosis Detection Kit (Becton Dickinson, San Jose, CA, USA) according to the manufacturer's instructions. Briefly, 1 × 10^6 ^U87 cells were harvested and washed with cold PBS. The cells were resuspended in 1 mL of 1 × binding buffer. One hundred microliters were transferred to a 5 mL culture tube, and 5 μL of Annexin V-FITC and 5 μL of propidium iodide (PI) were added. Cells were vortexed and incubated for 15 min in the dark. Four hundred microliters of 1 × binding buffer was added to each tube.

Flow cytometric analysis was performed immediately after staining. Data acquisition and analysis were performed by a fluorescence-activated cell scanner (FACS) flow cytometer (Becton Dickinson, San Jose, CA, USA). Cells in the early stages of apoptosis were Annexin V-positive and PI-negative, whereas cells in the late stages of apoptosis were positive for both annexin V and PI. All assays were independently repeated ≥ 3 ×.

### Tube formation assay

Cells growing in log phase were treated with trypsin and resuspended as single-cell solutions. A total of 2 × 10^5 ^HUVEC cells were seeded on Matrigel-coated 96-well plates. The cells were incubated with U87 supernatant that had been treated with null, Ad-vectors (MOI = 100), Ad-CALR vectors (MOI = 100) or Ad-CALR/MAGE-A3 vectors (MOI = 100) at 37°C, 5% CO_2 _for 48 h. Tube formation was quantified by counting the number of connected cells in randomly selected fields (×100). All assays were independently repeated ≥ 3 ×.

### Nude mouse xenograft model

Female BALB/c nu/nu mice, 4-5 weeks old, were purchased from Vital River Laboratories (Beijing, China). Animal treatment and care were in accordance with institutional guidelines. U87 cells (1 × 10^7^) were suspended in 100 μL PBS and injected subcutaneously into the right flank of each mouse. After 2 weeks, the tumor volume had reached 50-100 mm^3 ^and mice were randomly divided into four groups (n = 5 per group). The control group was left untreated. The 3 treatment groups consisted of the Ad-vectors, Ad-CALR and the Ad-CALR/MAGEA-3. Each of the mice in the 3 treatment groups were injected intratumorally with 100 μL of the respective treatment once every 7 days, for a total of 5 injections. The tumor diameters were measured 2 times per week with a caliper. The tumor volume (mm^3^) was calculated as: (length × width^2^)/2. All mice were euthanized humanely after 5 treatments, and the resected tumors were weighed.

### Statistical analyses

Statistical analyses were performed using Statistical Package for the Social Sciences version 16.0 software (SPSS, Chicago, IL). Data were expressed as mean ± standard deviation (SD), and analyzed using the Q-test or analysis of variance (ANOVA). The level of significance was set at *P *< 0.05.

## Results

### Identification of MOI in glioblastoma cell line U87

**Figure 1:**
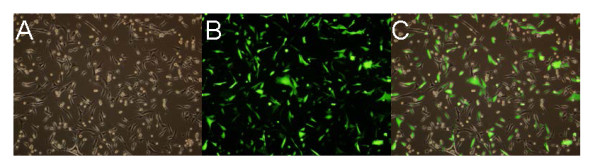
**Identificcation of MOI in glioblastoma cells**. Detection of MOI by fluorescence microscopy. A: under ordinary light; B: under fluorescence light; C: superimposed image of the two images. Optimal MOI of transfection with Ad-EGFP (green) in U87 cells were easily identified for 48 h post-transfection (×100).

To verify the transfection efficiency of Ad-vector in U87 cells, uptake of fluorescently-labeled Ad-vector (MOI 50, 100, 200) was detected by fluorescence microscopy 24 and 48 h after transfection. The test showed high-efficiency transfection: > 90% of cells displayed green fluorescence 48 h after transfection with 100 MOI Ad-enhanced GFP (EGFP; Figure 1).

### Expression of CALR and MAGE-A3 is examined by PCR and Western blot

**Figure 2:**
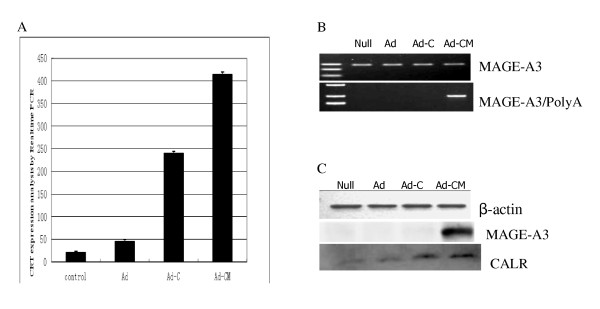
**Transfection of Ad-CALR/MAGE-A3 into glioblastoma cells**. (A): Comparision of expression of CALR in each group of U87 cells by quantitative RT-PCR. (B): Identification of expression of MAGE-A3 and MAGE-A3/PolyA by RT-PCR. (C): Identification of expression of CALR and MAGE-A3 in each grou of U87 cells by Western blotting.

To testify to the expression of CALR and MAGE-A3 and examine the differences among the four treatment groups, RT-PCR, qRT-PCR and Western blot were performed. The results of qRT-PCR showed that there were differences in CALR gene expression in U87 cells among the treatment groups. U87 transfected with Ad-CALR or Ad-CALR/MAGE-A3 expressed higher levels of CALR (Figure 2A). The results of RT-PCR showed that MAGE-A3 was expressed in each treatment group of U87 cells (Figure 2B). However, the transfection of MAGE-3A in U87 cells, demonstrated by the expression of MAGE-A3/PolyA, was demonstrated only in the Ad-CALR/MAGE-A3-transfected group (Figure 2B). Results of the Western blot indicated that CALR and MAGE-A3 protein was expressed in U87 cells of all treatment groups (Figure 2C).

### Inhibition of cell proliferation

**Figure 3:**
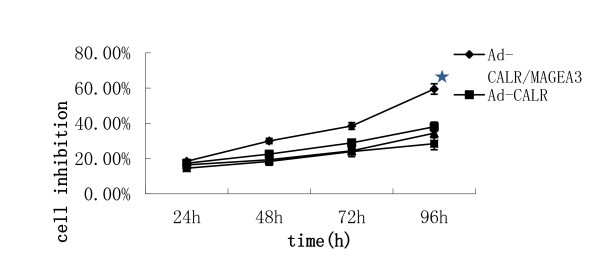
**Transfection of Ad-CALR/MAGE-A3 inhibited cell proliferation of glioblastoma cells in vitro**. Ad-CALR/MAGE-A3 transfected U87 cell growth was significantly attenuated in a time-dependent manner compared with control, Ad and Ad-CALR group. *P < 0.01.

The effect of Ad-CALR/MAGE-A3 transfection on glioblastoma cell proliferation was determined by MTT assay. The inhibition of cell proliferation was calculated as one minus the optical density reading taken at 490 nm. During the post-transfection observation period of 24-96 h, the proliferation of U87 cells transfected with Ad-CALR/MAGE-A3 was significantly inhibited in a time-dependent manner beginning at 48 h, when compared with the null, Ad-vector and Ad-CALR groups (Figure 3).

### Attenuation of invasion ability in Ad-CALR/MAGE-A3-transfected cells

**Figure 4:**
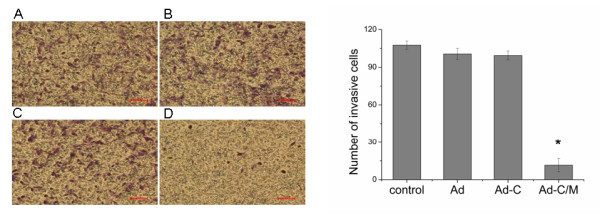
**Transfection of Ad-CALR/MAGE-A3 attenuated the invasion ability of glioblastoma cells in vitro**. Using matrigel coated invasion chambers, cell invasion ability was observed. The invading cells were fixed with cold methanol, and then stained with crystal violet. Representative microscopy images of the invasion assay are shown (×100). (A) - (D):Photomicrographs showing representative views of cell invasion assays. In the presence of Ad-CALR/MAGE-A3, the number of invading U87 (D) was smaller than those of U87 (A), U87/Ad-vector (B) cells and U87/Ad-CALR(C). Scale bars = 100 μm. (E): Bar represents the mean number of the cells per field. The invasion assay was consistent with the migration assay and showed that the transfection of Ad-CALR/MAGE-A3 attenuated the invasion ability of glioblastoma cells. *p < o.o5.

Tumor cell invasion is the critical step in the metastatic process. To verify the effect of Ad-CALR/MAGE-A3 on invasion ability, U87 cells were assayed using Transwell chambers pre-coated with Matrigel. After 48 h incubation, the invasive potential of Ad-CALR/MAGE-A3-transfected U87 cells was significantly suppressed, compared with the other groups (Figure 4). These results suggested that Ad-CALR/MAGE-A3 transfection attenuated the metastatic potential of glioblastoma cells in vitro.

### Flow cytometry indicate non-apoptotic effect on U87 of Ad vectors

**Figure 5:**
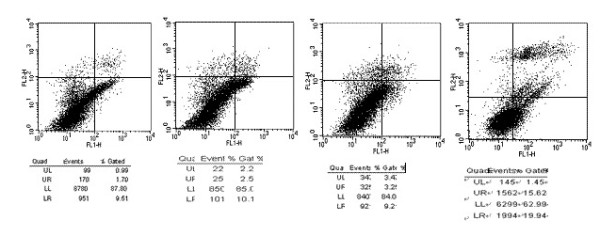
**Transfection of Ad-CALR/MAGE-A3 induced apoptosis of glioblastoma cells**. The transfected cells, labeled with AnnexinV-FITC and PI, were subjected to floe cytometric analysis. Two parameter histogram Dot Plot displayed FL1-FITC on the x axis and FL2-PI on the y axis. The result showed that Ad-CALR/MAGE-A3 increased the apoptotic rate in U87 cells.

To evaluate further whether Ad-mediated transfer of the genes of interest induced apoptosis in transfected U87 cells, 48 h after transfection cells were harvested and analyzed by flow cytometry. The rates of apoptosis of the null, Ad-vector, Ad-CALR and Ad-CALR/MAGE-A3 groups were 10.50%, 15.28%, 12.68% and 21.39%, respectively, and demonstrated that Ad-CALR/MAGE-A3 inducing apoptosis effect (Figure 5).

### Inhibition of tube formation in human umbilical vein endothelial cells

**Figure 6:**
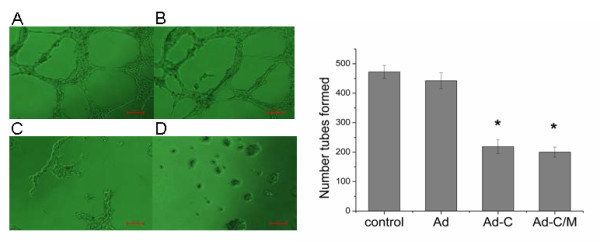
**Effect of Ad-CALR/MAGE-A3 on anti-angiogenesis in vitro**. Using matrigel coated 96 well plates, anti-angiogenesis ability was observed. (A) - (D): Photomicrographs showing representative views of tube formation assays. In the presence of Ad-CALR(C) or Ad-CALR/MAGE-A3(D), the number of connecting HUVEC was smaller than those of Null (A) and Ad-vector (B). Scale bars = 100 μm. (E): Bar represents the mean number of the cells per field. The tube formation assay showed that the transfection of Ad-CALR/MAGE-A3 attenuated the tube formation ability of HUVEC cells. Data are presented as mean ± SD (*P < 0.05, compared with HUVEC or HUVEC/Ad-VECTOR, *P *> 0.05, compared with HUVEC/Ad-CALR group).

Angiogenesis is the critical step in tumor initiation and progression. To determine the effect of Ad-CALR/MAGE-A3 on angiogenesis, tube formation in HUVEC cells was assayed. After treatment with the different supernatants for 48 h, the number of tubes formed was less in the Ad-CALR and Ad-CALR/MAGE-A3 groups than the other two groups (Figure 6). The ability of HUVEC cells to form tubes was significantly compromised by Ad-CALR/MAGE-A3. These data demonstrate that the antiangiogenic effect of transfection with combined CALR and MAGE-A3 was similar to that of transfection with CALR only.

### Molecular mechanisms underlying the antitumor effects of Ad-CALR/MAGE-A3

**Figure 7:**
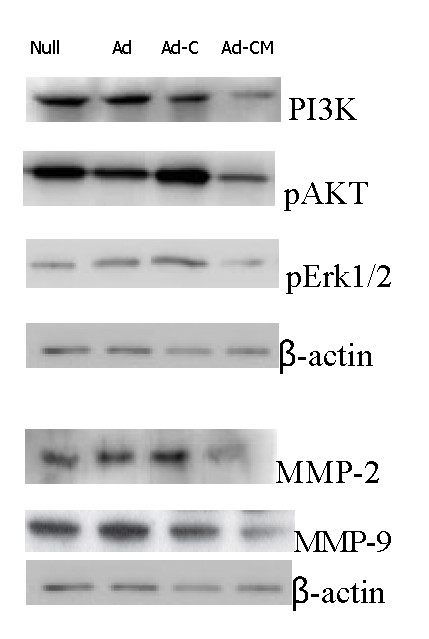
**Western blot analysis of PI3K/AKT**、**Erk1/2 and MMP-2/-9 by transfecting with Ad-CALR/MAGE-A3 in glioblastoma cells in vitro**. Representative images were shown. Expression of **PI3K/AKT**、**Erk1/2 and MMP-2/-9 **in Ad-CALR/MAGE-A3 group was significantly suppressed compared to that in other groups.

The protein from transfected cells was extracted to examine the effects of Ad-CALR/MAGE-A3 on some important cytokines and signaling molecules. After 48 h of transfection, the relative expression levels of the proteins PI3K, p-Akt, and p-Erk1/2 in the Ad-CALR/MAGE-A3 group were decreased, while there were no differences in the Ad-vector and Ad-CALR groups. The reduction was more significant after 96 h of transfection (Figure 7). Furthermore, compared to other groups, transfection with Ad-CALR/MAGE-A3 suppressed MMP2 and MMP9 expression (Figure 7). These data demonstrated that transfection with Ad-CALR/MAGE-A3 may inhibit signal transducer and activator of transcription (STAT)3, MMP2, and MMP9, which all play an important role in tumor progression.

### Inhibition of tumor growth of glioblastoma cells in nude mice by Ad-CALR/MAGE-A3

**Figure 8:**
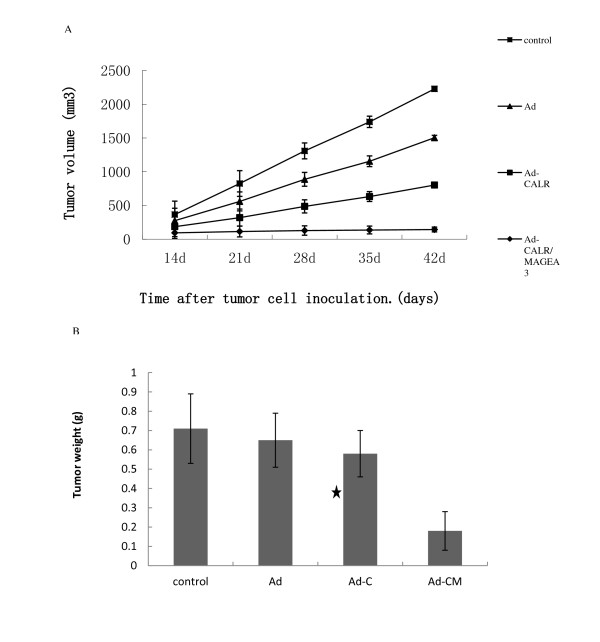
**Tumor volume curve and bar graph of tumor weight on the 42nd day when mice were killed**. (A): The curve showed that the tumor growth of Ad-CALR/MAGE-A3 group from days 25 to the end was significantly inhibited compared to that of control, Ad and Ad-CALR groups. (B): Bar represented that the tumor weight of Ad-CALR/MAGE-A3 group was decreased than that of control, Ad and Ad-CALR groups. **P < 0.01 versus other groups.

**Figure 9:**
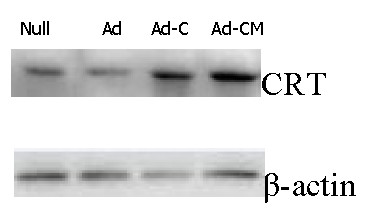
**Ad-CALR/MAGE-A3 reinforced the protein expression of CALR in vivo as determined by Western blot**. Representative images were shown. Expression of CALR in Ad-CALR/MAGE-A3 group was significantly reinforced compared to that in other groups.

Intra-tumoral injection with adenoviral vectors was performed to investigate whether Ad-CALR/MAGE-A3 had the effect of inhibition on tumor growth in vivo. A nude-mouse xenograft model of human glioblastoma was established, and when the tumor volume reached 50-100 mm^3^, intra-tumoral treatment with Ad-vectors were started and repeated every 7 days for a total of 5 injections. The mean tumor volume of the Ad-CALR/MAGE-A3 group from day 25 to the end was significantly smaller than that of the other groups, whereas there was no statistical differences among the other groups throughout the experimental period (Figure 8A). All mice were euthanized on the 42^nd ^day, and the final tumor volume and weight in the Ad-CALR/MAGE-A3 group (142.6 ± 84.2 mm^3 ^and 0.18 ± 0.10 g, respectively) were markedly smaller than in the null (724.2 ± 198.4 mm^3 ^and 0.71 ± 0.18 g), Ad-vector (701.4 ± 183.2 mm^3 ^and 0.65 ± 0.14 g) and Ad-CALR (659.2 ± 147.8 mm^3 ^and 0.58 ± 0.12 g) groups (n = 5, each group; Figure 8B). In addition, the relative protein expression of CALR in the Ad-CALR/MAGE-A3 group was increased significantly (Figure 9). Altogether, these results indicate that intratumoral injection with Ad-CALR/MAGE-A3 suppressed the tumor growth of glioblastoma cells in vivo.

## Discussion

Glioblastoma is the most common and aggressive form of brain tumor that affects adults. Despite advances in surgical and clinical neuro-oncology, the prognosis for glioblastoma remains poor due to its diffuse and invasive nature [24]; tumor cells are highly proliferative and invasive within the brain. Tumor progression involves tumor cell proliferation and invasion, vascular intravasation and extravasation, establishment of a metastatic niche, and angiogenesis [25-27]. Therefore, to improve outcome the focus of gene therapy strategy is to effectively inhibit the proliferative, invasive, and angiogenic behavior of glioblastoma cells.

Studies have shown that CALR plays an important role in the biological processes of many cancers, and these mechanisms are mediated via antiangiogenic factors and the immune response. There is wide recognition that in glioblastoma, CALR expression is increased, with high radiation sensitivity [28]. However, a definite conclusion that the expression of CALR with MAGE-A3 in glioblastoma affects tumor cell proliferation, apoptosis, and invasion processes has not been established.

In order to evaluate the effect of Ad-CALR/MAGE-A3 on U87 glioblastoma cells, we over-expressed human CALR and MAGE-A3 in U87 cells via adenovirus-mediated gene transduction, ensuring that we used the appropriate number of PFUs (MOI = 100) to obtain high expression of CALR and MAGE-A3. The present in vitro study demonstrated that the proliferative and invasive properties of cells transfected with Ad-CALR/MAGE-A3 were attenuated in comparison to the other treatment groups and controls. The in vivo study demonstrated that the tumor formation rate, and the average tumor growth rate, of the Ad-CALR/MAGE-A3-transfected group was significantly lower than that of the non-transfected, Ad-vector-transfected and Ad-CALR-transfected groups. Thus, these results suggest that an Ad-vector encoding CALR chimerically linked to MAGE-A3 is a unique approach for the generation of a potent antitumor effect.

In the current study, CALR and MAGE-A3 overexpression in glioblastoma cells suppressed the Erk1/2 MAPK and PI3K/Akt signal pathways, which are well recognized for mediating cell proliferation and apoptosis. This result explains, at least in part, why Ad-CALR/MAGE-A3 inhibited cell proliferation and induced apoptosis in U87 cells.

Furthermore, the expressions of MMP2 and MMP9 were downregulated in Ad-CALR/MAGE-A3- transfected cells, and this may suggest that these MMPs are the downstream products of CALR and MAGE-A3-induced cell signaling. MMPs are a family of enzymes that degrade proteins in the extracellular matrices of tissues, and are clearly involved in stages of cancer progression, including tumor cell degradation of basement membranes and stroma, and blood vessel penetration [29,30]. Consequently, the reduction of MMP2 and MMP9 by Ad-CALR/MAGE-A3 will attenuate the metastatic potency of glioblastoma cells. Ad-CALR/MAGE-A3 also generated a therapeutic effect due to inhibition of angiogenesis.

Tumor growth and metastasis formation depends on an adequate blood supply. As neoplasms grow larger, blood supply to the tumor is often ensured by new vessel formation, a process termed angiogenesis. Therapeutic agents that target tumor vasculature may prevent or delay tumor growth and even promote tumor regression or dormancy [31,32]. Previous studies demonstrated the CALR and its protein fragment (aa 1-180) vasostatin are endothelial cell inhibitors of tumor growth [33,34]. Therefore, gene therapy employing CALR may enhance antitumor responses and antiangiogenic effects. In the present study, the tube formation assay showed that Ad-CALR/MAGE-A3 attenuated the angiogenic potential of glioblastoma cells.

In this study, we constructed an innovative adenoviral vector Ad-CALR/MAGE-A3. Our results demonstrate that Ad-CALR/MAGE-A3 can significantly suppress the invasive potency of U87 cells. Furthermore, transfection with Ad-CALR/MAGE-A3 resulted in the inhibition of angiogenesis. Thus, adenoviral-mediated delivery of CALR chimerically linked to MAGE-A3 represents a unique approach for the generation of potent antitumor effects.

## Conclusions

In summary, our findings show for the first time that overexpression of CALR and MAGE-A3 in glioblastoma cells by Ad-CALR/MAGE-A3 transfection can inhibit tumor growth and invasion in vitro and in vivo. Furthermore, these antitumor effects may be associated with antiangiogenesis in glioblastoma. Therefore, Ad-CALR/MAGE-A3 may potentially be a useful tool for gene therapy of glioblastoma, and even other cancers.

## Competing interests

The authors declare that they have no competing interests.

## Authors' contributions

XLL and PM designed the study. XLL, DZ, YW, FQQ, DPS, and YL performed the experiments. XLL drafted the manuscript. PM supervised the experimental work. All authors read and approved the final manuscript.
